# Pedicled Anterolateral Thigh Flap (ALT) for Vulva Reconstruction: Journey to a New Horizon

**DOI:** 10.1007/s13224-024-02060-x

**Published:** 2024-10-01

**Authors:** Dimpy Begum, Debabrata Barmon, Upasana Baruah, Shruti Darak, R. Aparajita, Mahendra Kumar, Karthik Chandra Bassetty

**Affiliations:** 1 Gynaecological Oncology, Dr B.Borooah Cancer Institute, Guwahati, India; 2 Department of Plastic Surgery, Tata Medical Hospital, Mumbai, India

**Keywords:** Vulval cancer, V–Y plasty, ALT flap, Plastic surgery

## Abstract

Surgery for vulval malignancies necessitates extensive resection necessitating the use of musculocutaneous grafts. We present one such case where the patient needed both a V–Y plasty and an anterolateral thigh flap to ensure a tension-free repair. She underwent a partial deep vulvectomy followed by V–Y plasty and ALT flap. The procedure has been described in detail along with intraoperative photographs. She recovered uneventfully and is receiving post-operative adjuvant radiotherapy. We would like to highlight this “additional skill” of plastic surgery which will empower surgeons to undertake radical surgeries to ensure the best outcomes for patients.

## Introduction

Vulval cancer is a rare disease accounting for 1–3% of all female genital tract malignancies. [1] Many patients do not seek medical help until late in the course of the illness. The majority of women undergo radical vulvectomy and inguinofemoral lymphadenectomy.

Radical vulvectomy can create a large defect of the vulva which cannot be closed by primary closure or by rotation grafts. We report one such case of carcinoma vulva which necessitated the use of the anterolateral thigh (ALT) flap post-vulval tumour excision.

## Case Report

A 27-year-old multiparous woman presented to Dr. Bhubaneswar Borooah Cancer Institute, Guwahati, Assam, with complaints of perineal itching and swelling in the genital region for the past one year.

**Fig. 1:**
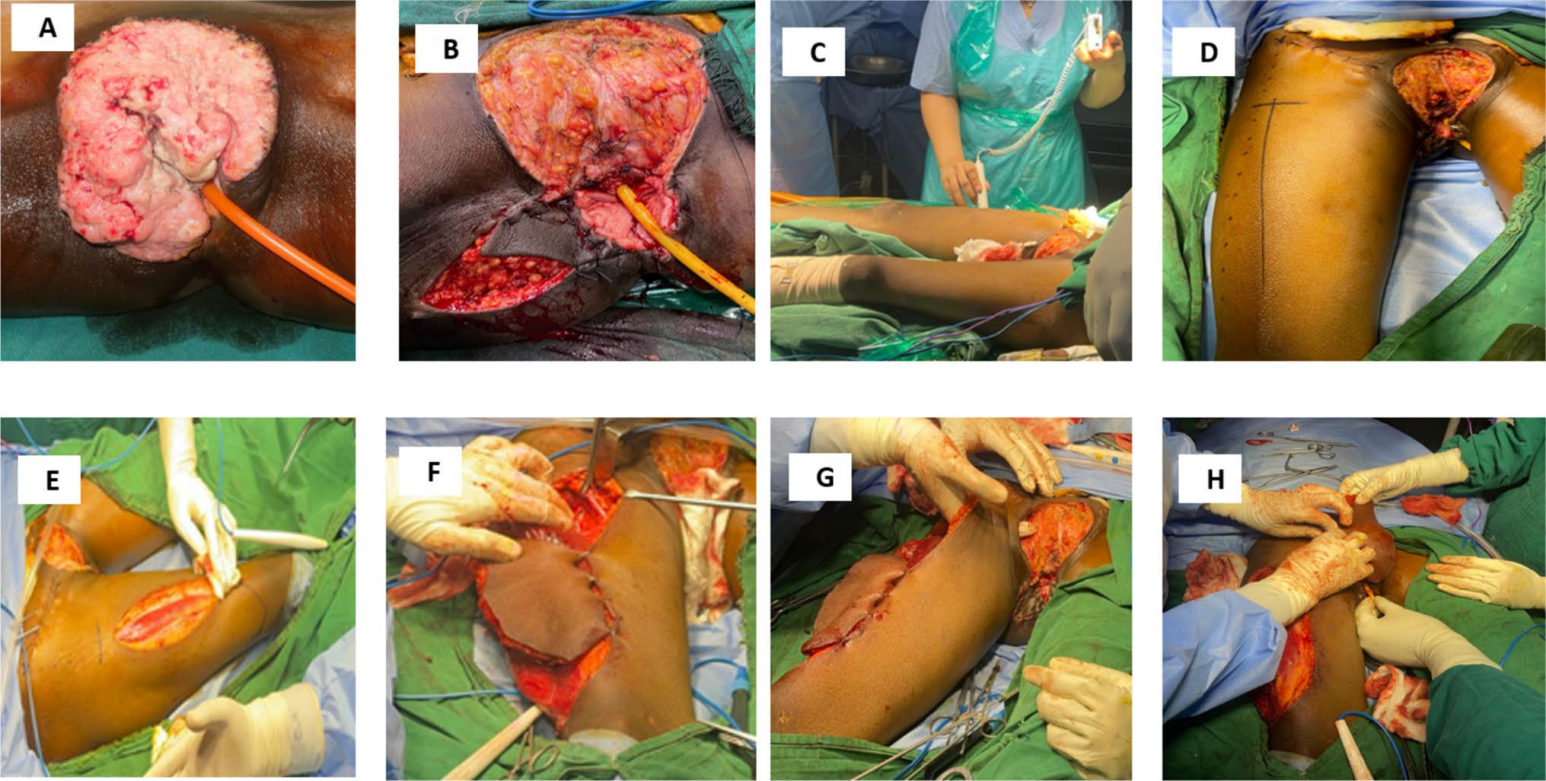
Intraoperative procedure of **A** preoperative photograph, **B** wide perineal defect and right V–Y plasty, **C** Doppler assessment of perforators, **D** skin marking, **E** raising of ALT flap, (F G H) raising of rectus femoris/sartorius and tunnelling to the perineum

On examination, as shown in Fig. 1A there was a large firm proliferative growth of size 11*7*5 cm lesion involving bilateral labia majora and minora. The lesion involved the clitoris and was 0.5 cm away from the urethra. The lower edge of the lesion was 2 cm away from the anal margin. It was abutting the margin of the vagina bilaterally. Tenderness was noted due to the extensive size of the lesion. Bilateral inguinofemoral lymph nodes were 2 cm in size, firm and mobile in nature. Liquid-based cytology of the cervix was suggestive of negative for intraepithelial lesion or malignancy (NILM). Vulval biopsy showed well-differentiated squamous cell carcinoma. Contrast-enhanced magnetic resonance imaging (MRI) showed a 7*6 cm ulceroproliferative growth abutting the urethral orifice and left lateral wall of the vagina. Fine needle aspiration cytology (FNAC) from the inguinal nodes was suggestive of metastatic squamous cell carcinoma.

She underwent total deep vulvectomy and bilateral inguinofemoral lymph node dissection as shown in Fig. 1. Using a margin of 1 cm, the entire lesion including bilateral labia majora and the distal 1 cm of the urethra were excised. Post-excision of the mass a large defect of 14*10 cm was present. To reduce the defect size, a V–Y plasty was done on the right side as shown in Fig. 1. Further a residual defect of size 10*8 was left, and a decision to perform a right ALT flap was made.

**Fig. 2:**
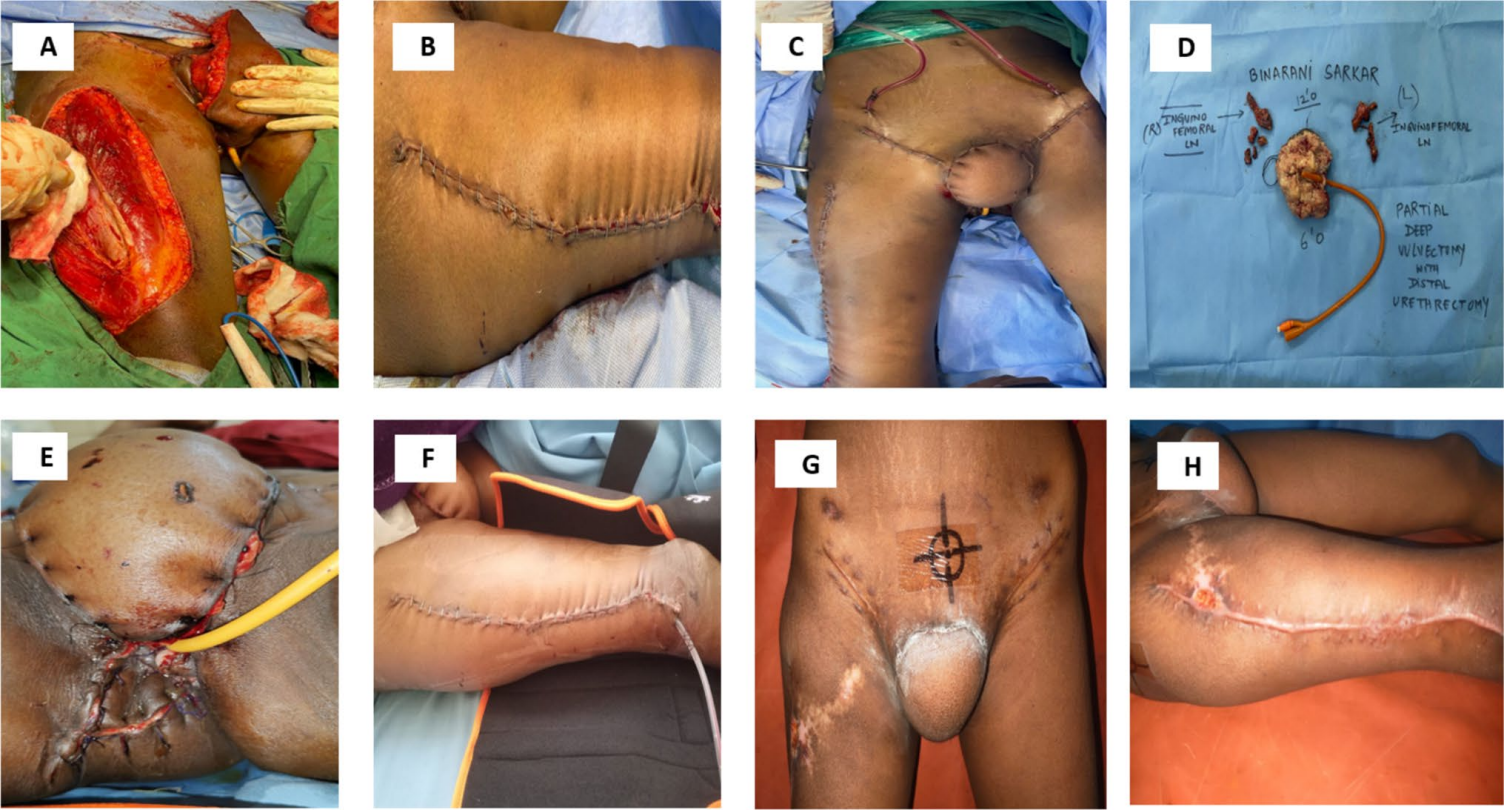
**A**, **B** Donor site defect and primary closure, **C**, **D** Immediate post-operative wound site and gross specimen, **E**, **F** Post-op day 2 vulval and leg site wound, **G**, **H** Post-op day 28 prior to adjuvant radiotherapy

The perforators for the anterolateral thigh flap which arise from the lateral femoral circumflex artery were marked using a handheld Doppler device. Surface marking was done with a straight line drawn from the anterior superior iliac spine to the central part of the patella. Planning in reverse was done for the defect, and the paddle was marked. The anterior incision was made, and the rectus femoris muscle was identified with its bipinnate nature. No perforator was identified at the required skin paddle. The decision was taken to raise a musculocutaneous flap based on vastus lateralis. The paddle consisting of skin and underlying cuff of vastus lateralis muscle along with the descending branch of the lateral circumflex femoral artery was raised. After skeletonizing the pedicle, the paddle along with the muscle was passed underneath the rectus femoris and sartorius muscles to the perineal defect. After ensuring adequate tunnelling and proper orientation of the pedicle, the flap inset was done. Flap bleeding was confirmed, and the donor site was closed primarily over a negative pressure suction drain as shown in Fig. 2. We used an above-knee brace to ensure the knee was extended in the post-operative period.

Post-operative the patient was administered intravenous antibiotics for five days and subcutaneous low molecular weight heparin prophylaxis for 2 weeks. The flap was daily monitored for colour changes, turgor, temperature and sensation. Physiotherapy, ambulation and a soft diet were started by day 2. The ALT drain was removed by day 2. On post-op day 8, there was minimal dehiscence of the VY flap at the vaginal margin. Regular dressing was done and following healing of the wound she was discharged on post-op day 11. The urinary catheter was placed for 3 weeks given the distal urethrectomy.

The final histopathological report showed squamous cell carcinoma with negative margins. However, as one inguinal node out of fourteen nodes removed was positive for malignancy, she was planned for adjuvant radiotherapy in the Disease Management Group meeting. The wound has completely healed, and she is undergoing radiotherapy.

## Discussion

The goal of vulval reconstruction surgeries includes tension-free wound closure, maintaining the introital opening and maintaining the orientation of urethral and anal opening to maintain a good quality of life. [2] The V–Y advancement flap either from the medial thigh or gluteal fold is based on the suprafascial vascular plexus from superficial/deep femoral arteries and the superficial perineal artery. The use of regional flaps carries a risk of delayed wound healing especially in the post-operative setting of adjuvant radiation therapy. The ALT flap is gaining rapid popularity given its versatility either as a pedicled form or as a free flap. [3] When the flap is harvested with the vastus lateralis as a myocutaneous flap, its single dominant vascular pedicle makes it a type I Mathes–Nahai myocutaneous flap.

The ALT flap is used to reconstruct wide defects in the inferior posterior trunk, inferior abdomen, ipsilateral groin, vagina and perineum. The dominant blood supply to the vastus lateralis and anterolateral skin is the descending branch of the lateral circumflex femoral artery. The 3D vascular structure of the ALT flap resembles a tree with the nutrient vessels arborizing in each layer with a progressive decrease in length and diameter of vessels from the deeper to the superficial layers. [4] It is important to preserve the blood supply to the rectus femoris and branches of the lateral femoral cutaneous nerve which provide the sensory innervation to the skin of the anterolateral thigh.

The advantages of an ALT flap are multifold: similar skin texture as the vulva, intact sensory nerve function, good function and shape to cover the vulval defect. The most critical process of any flap repair is to preserve the flap’s vascular pedicle and to ensure non-tension closure of wounds. The donor site might require a split-thickness skin graft. ALT flap has the added advantage of minimal donor site morbidity.

The novelty of this case report lies in the combined use of a V–Y plasty and ALT flap. It highlights the importance of having a plastic surgeon on board when we anticipate extensive residual vulval defect following radical vulvectomy.

## Conclusion

ALT flap is a versatile flap that finds its rightful place in the armamentarium of a gynaecologic oncologist to achieve tension-free wound closure in radical vulval surgeries.

## Data Availability

This study encompasses all data generated or scrutinized within its scope, comprehensively integrated within this case report.
